# Supplementary material for: Cell Penetrating Peptide-Based Self-Assembly for PD-L1 Targeted Tumor Regression
Source: Int J Mol Sci. 2021 Dec 10;22(24):13314. doi: 10.3390/ijms222413314 (PMC8703959; doi:10.3390/ijms222413314)
Supplement: Supplementary file 1 [file ijms-22-13314-s001.zip › ijms-1502704-supplementary.pdf]

International Journal of
Molecular Sciences

MDPI

Article

Cell Penetrating Peptide-Based Self-Assembly for PD-L1 Targeted Tumor Regression

Feng Guo [1,2,3], Junfeng Ke [1,2,3], Zhengdong Fu [1,2,3], Wenzhao Han [1,2,3] and Liping Wang [1,2,3,*]

Supplementary data

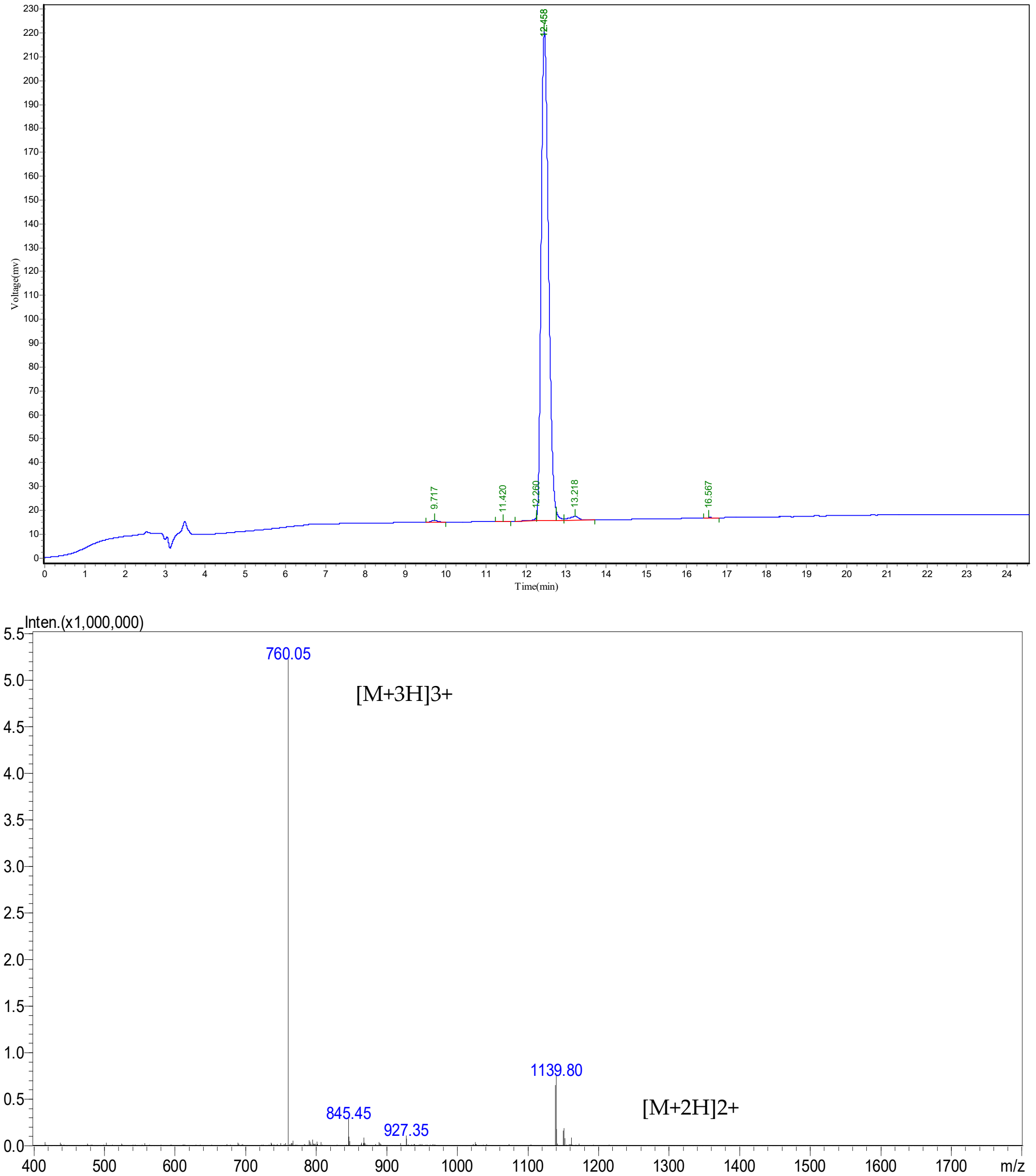

Figure S1. High-performance liquid chromatography (HPLC) spectrum and mass spectrometry of peptide. HPLC was performed with 73% (0.1% TFA) water and 27% acetonitrile (0.1% TFA), which indicated that the purity of peptide was more than 97.4%. Mass spectrometry showed the main peaks of peptide with 3 or 2 H^+, proving the relative molecular weight to be 2277.74. The results illustrated the peptide were successfully synthesized.

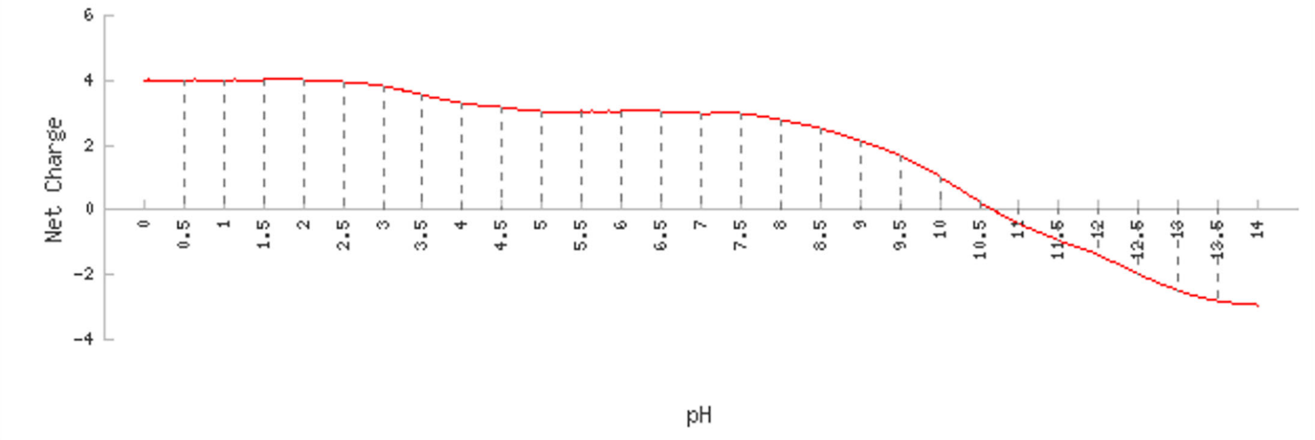

Figure S2. Net charge of peptide in aqueous solution, calculated by the online tool 'Concentration polypeptide property calculator' from NovoPro.

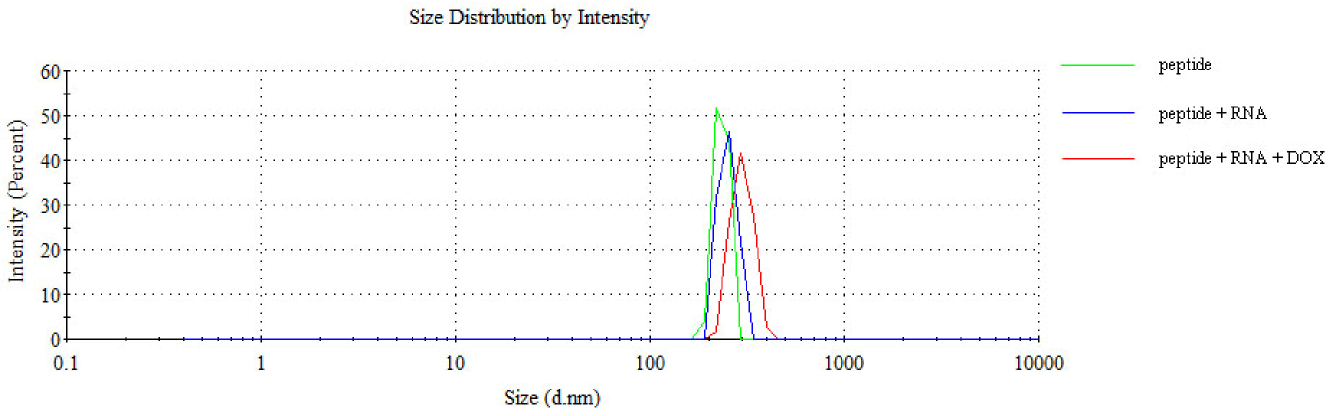

Figure S3. Zeta potential of peptide-nanovesicles detected by Malvern NANO ZS90 at the concentration from 0.0625 mg/mL to 0.5 mg/mL.

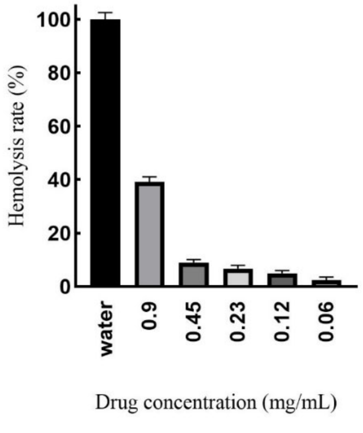

Figure S4. Hemolysis assay. Blood from SD rat were centrifuged and washed to get red blood cells. After incubation with different peptide and centrifugation, supernatant was detected at 545 nm.

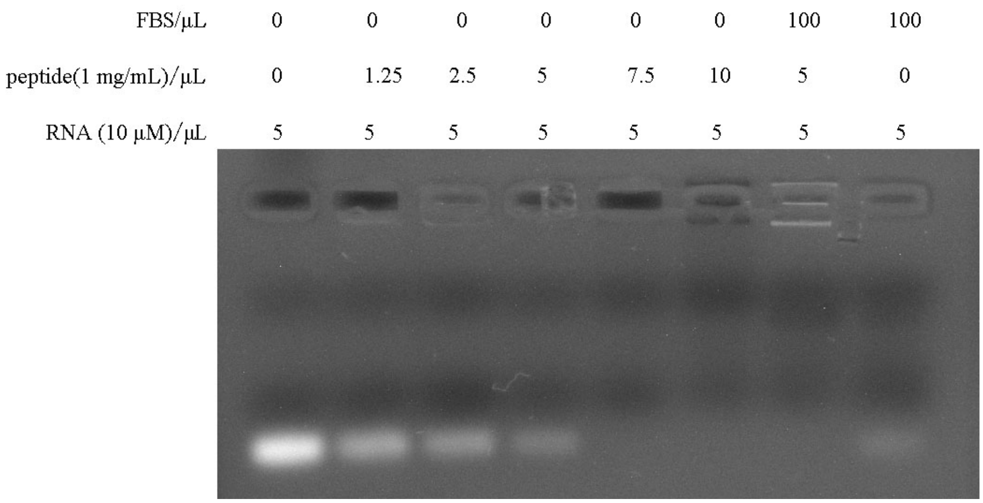

Figure S5. Gel retardation assays.

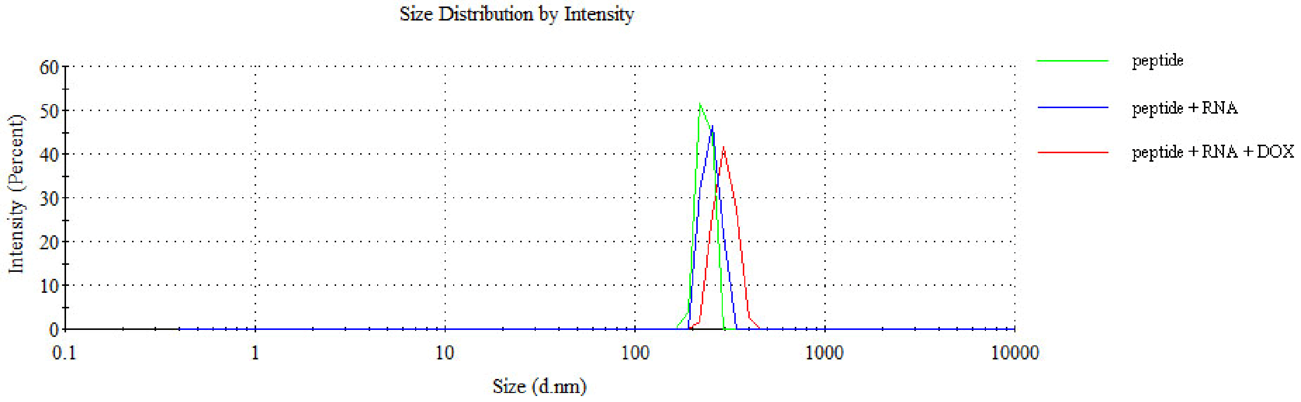

Figure S6. Sizes of peptide, peptide-RNA, peptide-RNA-DOX were detected by DLS.

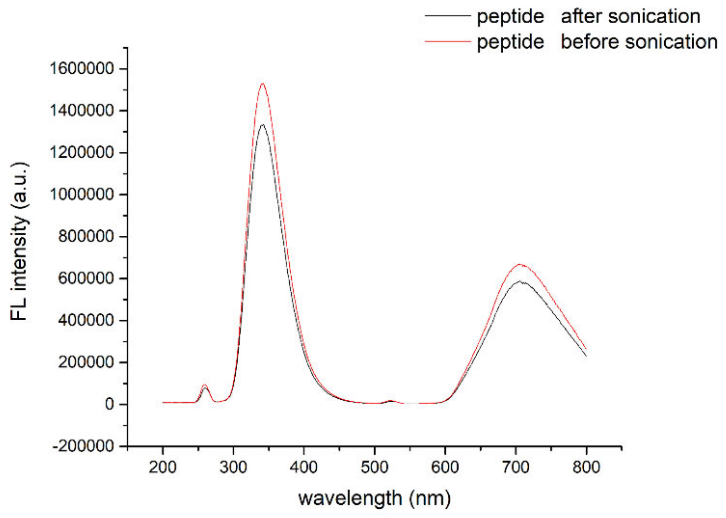

Figure S7. Fluorescence spectra of peptide before and after sonication.

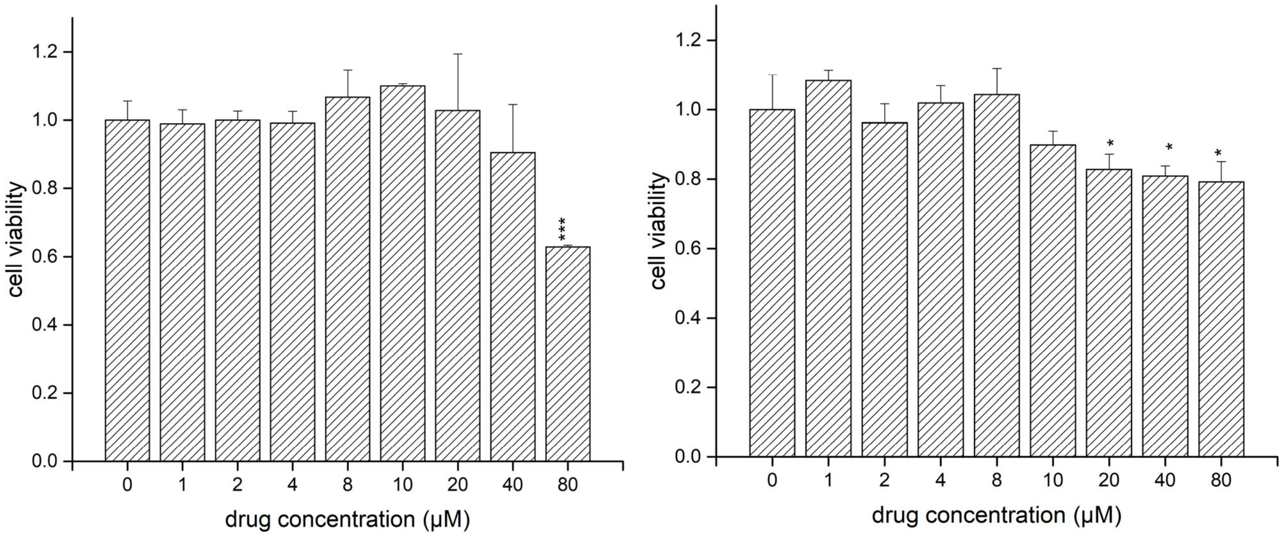

Figure S8. MTT assay carried with MDA-MB-231 (left) cells and HUVEC cells (right). *$p < 0.05$, **$p < 0.01$, ***$p < 0.001$, $n = 3$.

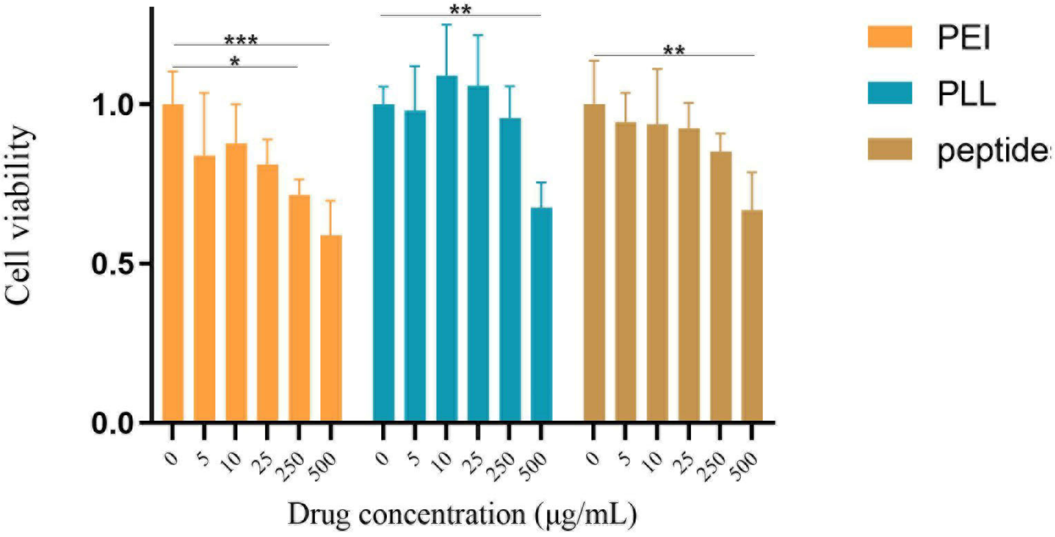

Figure S9. Peptide were compared to PEI and PLL. Their biocompatibilities were assessed by MTT assays. *$p < 0.05$, **$p < 0.01$, ***$p < 0.001$, $n = 3$.

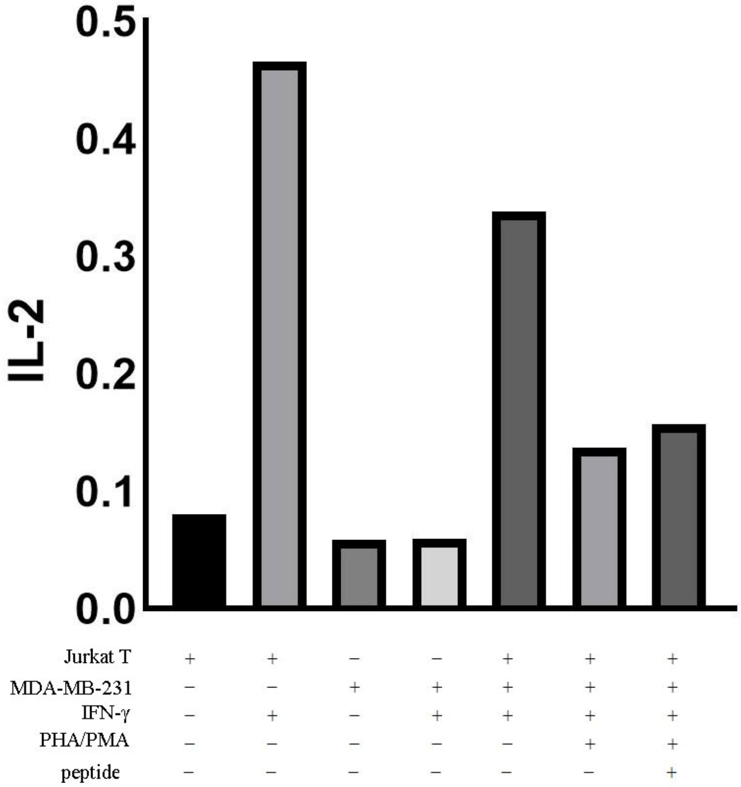

Figure S10. Peptide blockades the PD-1/PD-L1 process in vitro.

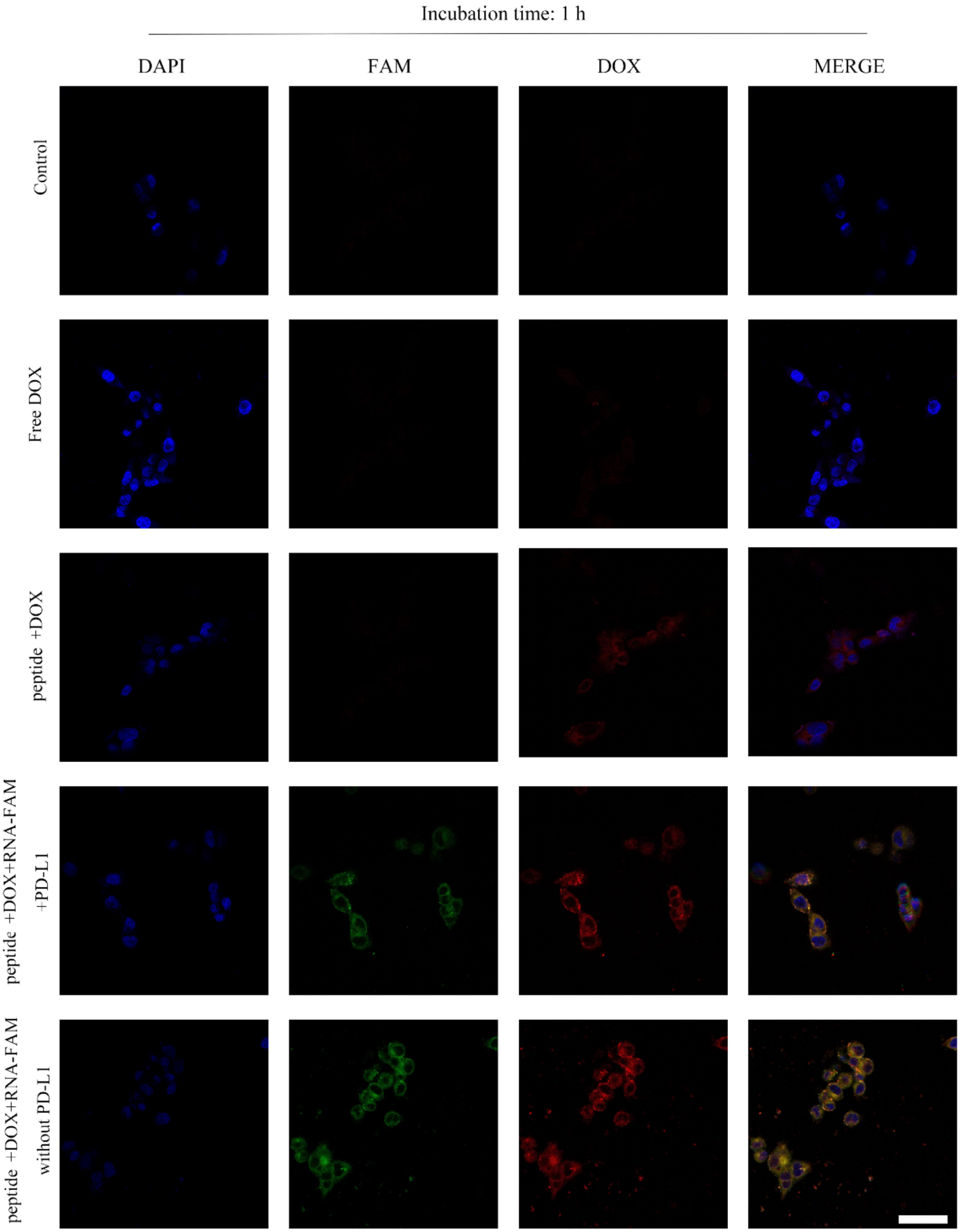

Incubation time: 1 h
DAPI
FAM
DOX
MERGE
Control
Free DOX
peptide +DOX
peptide +DOX+RNA-FAM
+PD-L1
peptide +DOX+RNA-FAM
without PD-L1

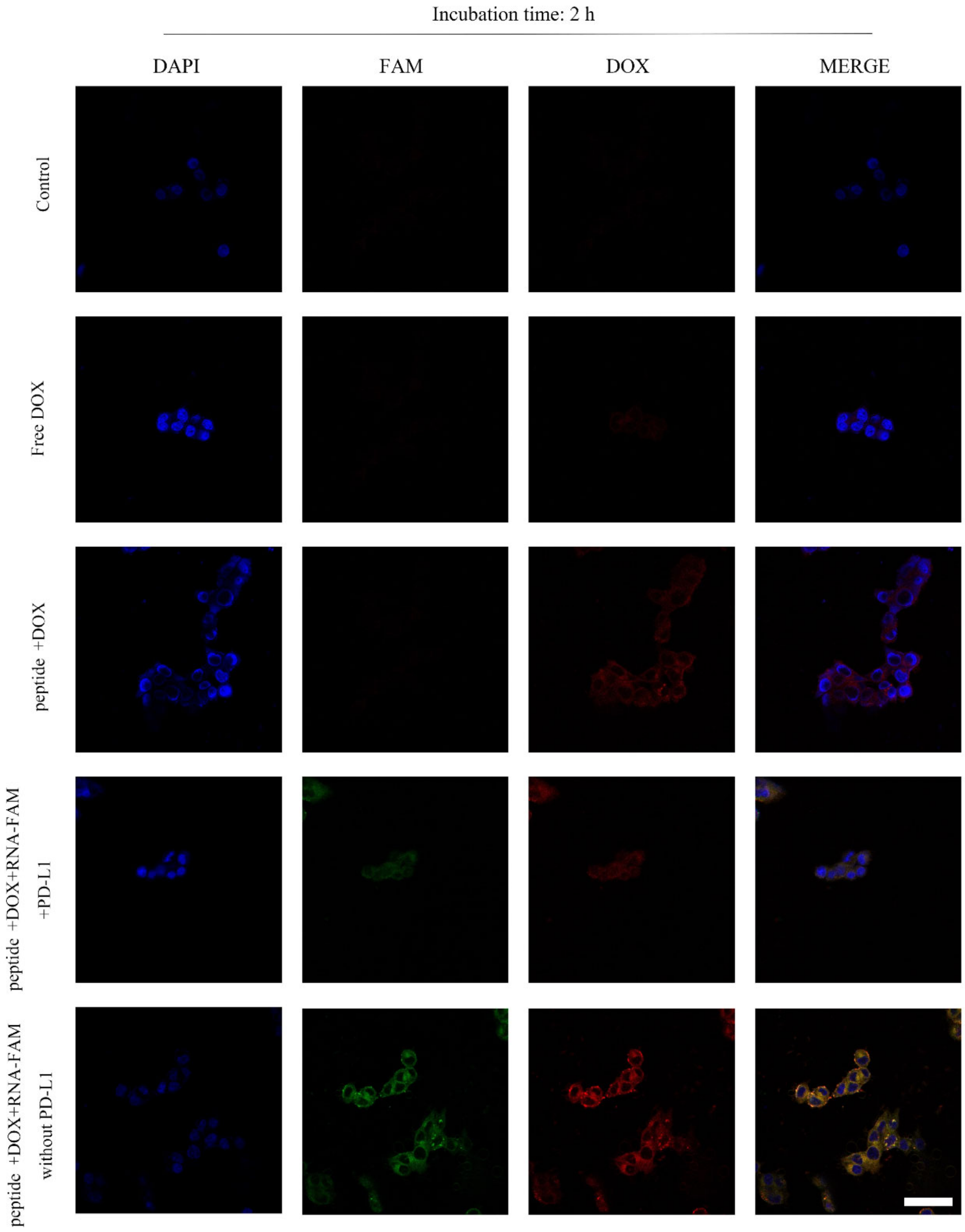
Incubation time: 2 h
DAPI
FAM
DOX
MERGE
Control
Free DOX
peptide +DOX
peptide +DOX+RNA-FAM +PD-L1
peptide +DOX+RNA-FAM without PD-L1

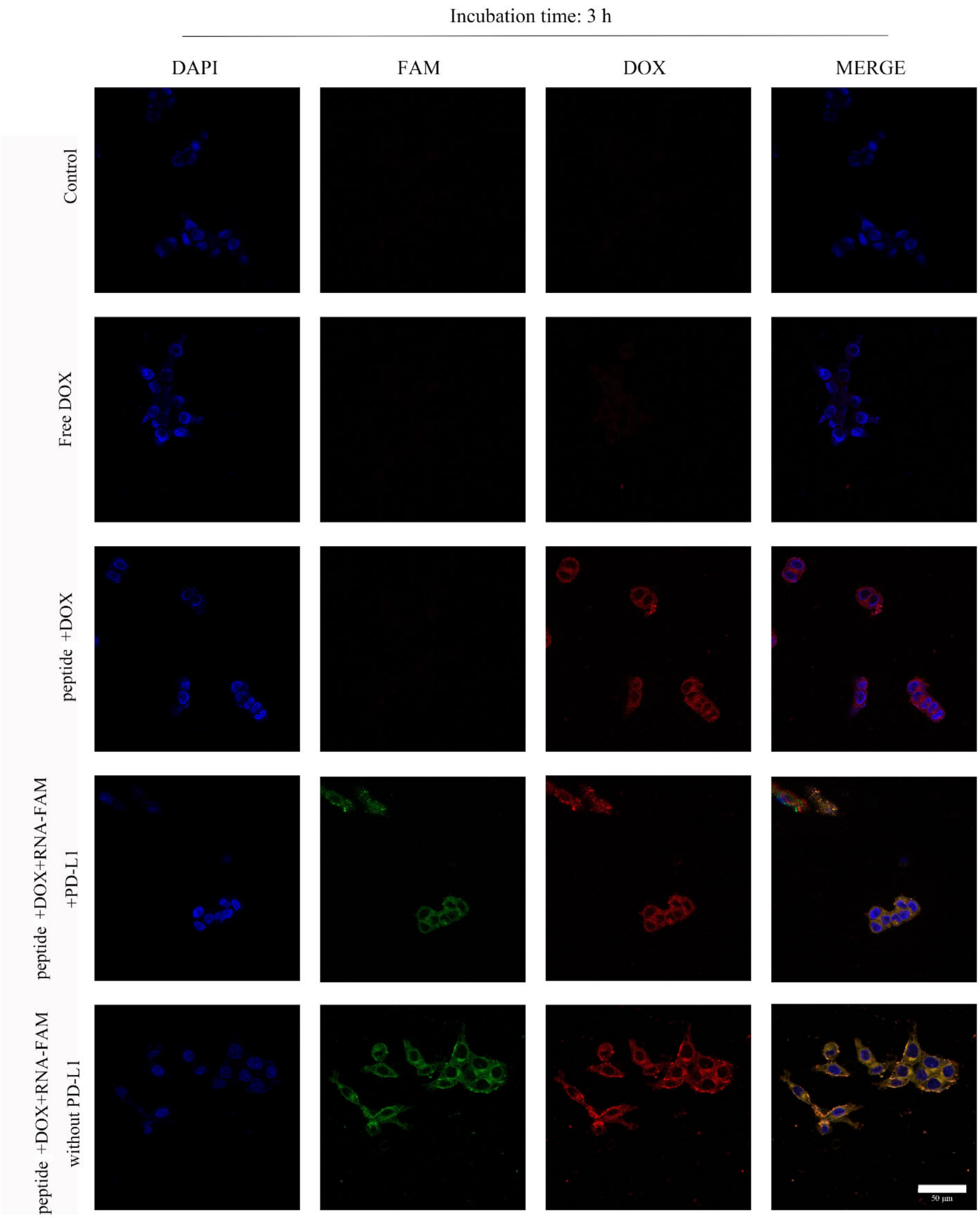

Figure S11. CLSM of peptide, peptide-RNA, peptide-RNA-DOX after 1h, 2h, 3h. CLSM images of cells after the incubation with PBS (the first line), free DOX (the second line), peptide-DOX (the third line), peptide-RNA-DOX with PD-L1 proteins (the fourth line), pep-tide-RNA-DOX (the fifth line). Scale bar 50 μm.

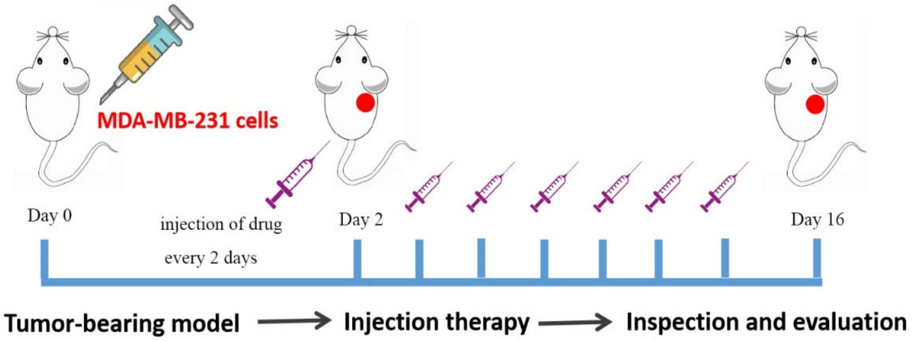

Figure S12. Illustration of the experimental flow chart of tumor-bearing mice. Tumor cells were injected 2 days before the administration of drugs. Then drugs were injected into the vicinity of tumor nods once every 2 days.

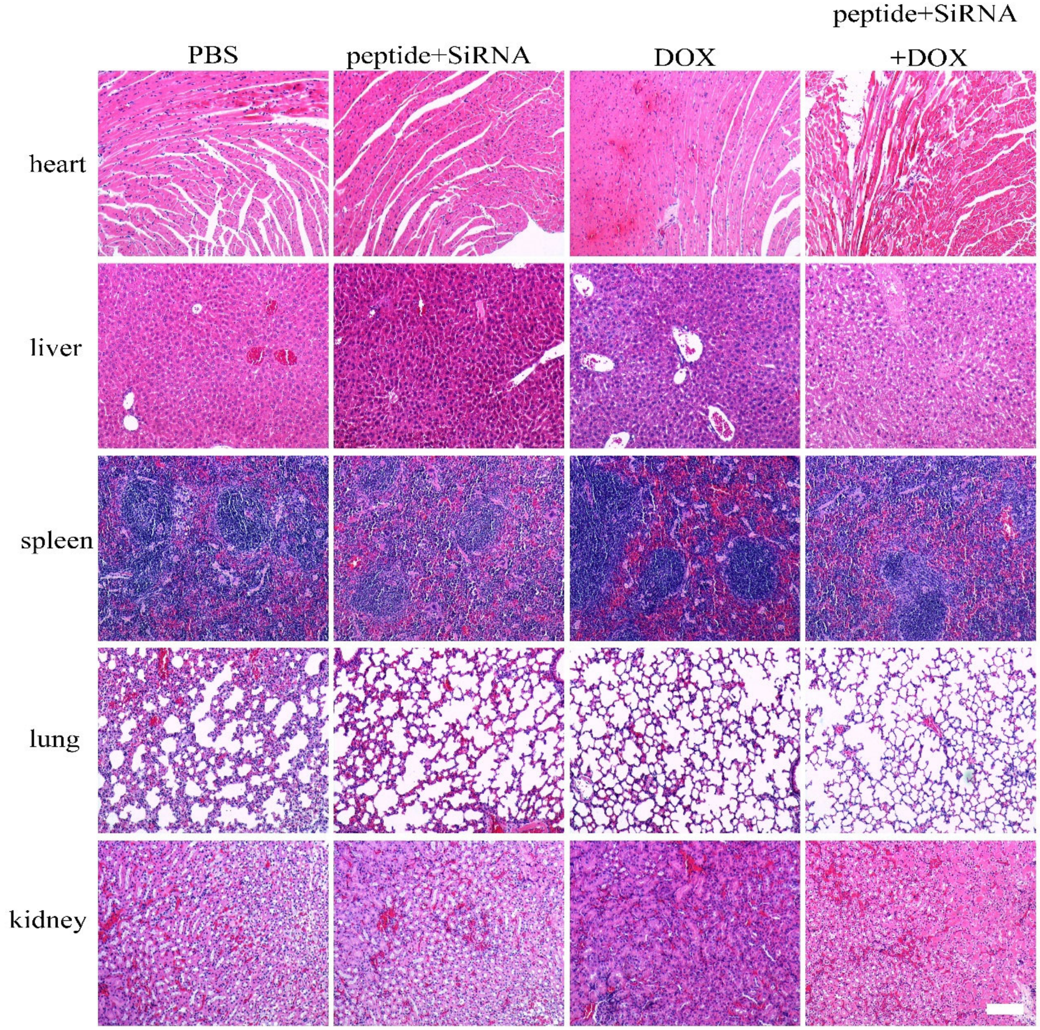

Figure S13. H&E staining of hearts, livers, spleens, lungs, and kidneys. Mice administrated with PBS, free DOX, peptide-RNA, and peptide-RNA-DOX were sacrificed 16 days after the treatment. Significant decrease in lung metastasis can be observed. Heart inflammation was obvious when using DOX. Scale bar. 200 μm.

Table S1. Actual drug loading (DLC) and drug loading rate (DLE) at theoretical dosage.ratios of 10%, 20%, and 30%.

	Theoretical DLC (wt%)	DLC (wt%)	DLE (wt%)
Peptide-DOX	10	10.1 ± 0.59	101.1 ± 5.9
	20	10.6 ± 0.75	106.3 ± 7.5
	30	7.2 ± 0.39	72.0 ± 3.9
Peptide-Nucleic acid-DOX	10	10.2 ± 0.60	102.2 ± 6.0
	20	10.4 ± 0.62	103.8 ± 6.2
	30	8.65 ± 0.26	86.5 ± 2.6
